# Importance of Arrhythmic Origin in the Diagnosis of Isolated Cardiac Sarcoidosis: A Case Report

**DOI:** 10.7759/cureus.73015

**Published:** 2024-11-04

**Authors:** Yuto Tsumuki, Hirofumi Kawamata, Masatoshi Hori, Daiki Shako, Tatsuya Kawasaki

**Affiliations:** 1 Department of Cardiology, Matsushita Memorial Hospital, Moriguchi, JPN; 2 Department of Emergency Medicine, Matsushita Memorial Hospital, Moriguchi, JPN

**Keywords:** cardiac sarcoidosis, computed tomography, echocardiography, isolated, ventricular tachycardia

## Abstract

Sarcoidosis has a heterogeneous clinical presentation and remains difficult to diagnose, especially in isolated cardiac sarcoidosis (CS). We report a case of life-threatening arrhythmia that led to the diagnosis of isolated CS. A 63-year-old man presented with sustained ventricular tachycardia that was thought to originate from the anterolateral free wall of the right ventricle. Electrocardiography, echocardiography, and coronary computed tomography performed after the return to sinus rhythm were initially considered unremarkable, but right ventricular free wall bulging was later noted on computed tomography. Follow-up echocardiography revealed abnormal wall motion not only in the right ventricular free wall but also in the left ventricular apex. The patient was finally diagnosed with isolated CS and was scheduled for steroid treatment after receiving an implantable cardioverter-defibrillator.

## Introduction

Sarcoidosis is a complex disease of unknown etiology with heterogeneous clinical presentation, such as various arrhythmias or heart failure, and a diagnosis of sarcoidosis, especially isolated cardiac sarcoidosis (CS), remains a clinical challenge [1,2]. We report a case of isolated CS with the development of sustained ventricular tachycardia. Initially, no abnormal findings were recognized on electrocardiography, echocardiography, and coronary computed tomography performed after recovery from the fatal arrhythmia, but later, a bulging of the right ventricular free wall was noticed on computed tomography, which led to the diagnosis of isolated CS.

## Case presentation

A 63-year-old man presented to the emergency department of our hospital with a 45-minute history of chest tightness that developed suddenly after a dull headache and later radiated to the jaw. His medical history was significant for hypertension, dyslipidemia, and hyperuricemia. His medications were irbesartan 50 mg daily, ethyl icosapentate 1800 mg daily, febuxostat 20 mg daily, and simvastatin 5 mg daily. He was an ex-smoker with a 20-pack-year history, drank moderately every day, did not use illicit drugs, and had no known allergies.

**Figure 1:**
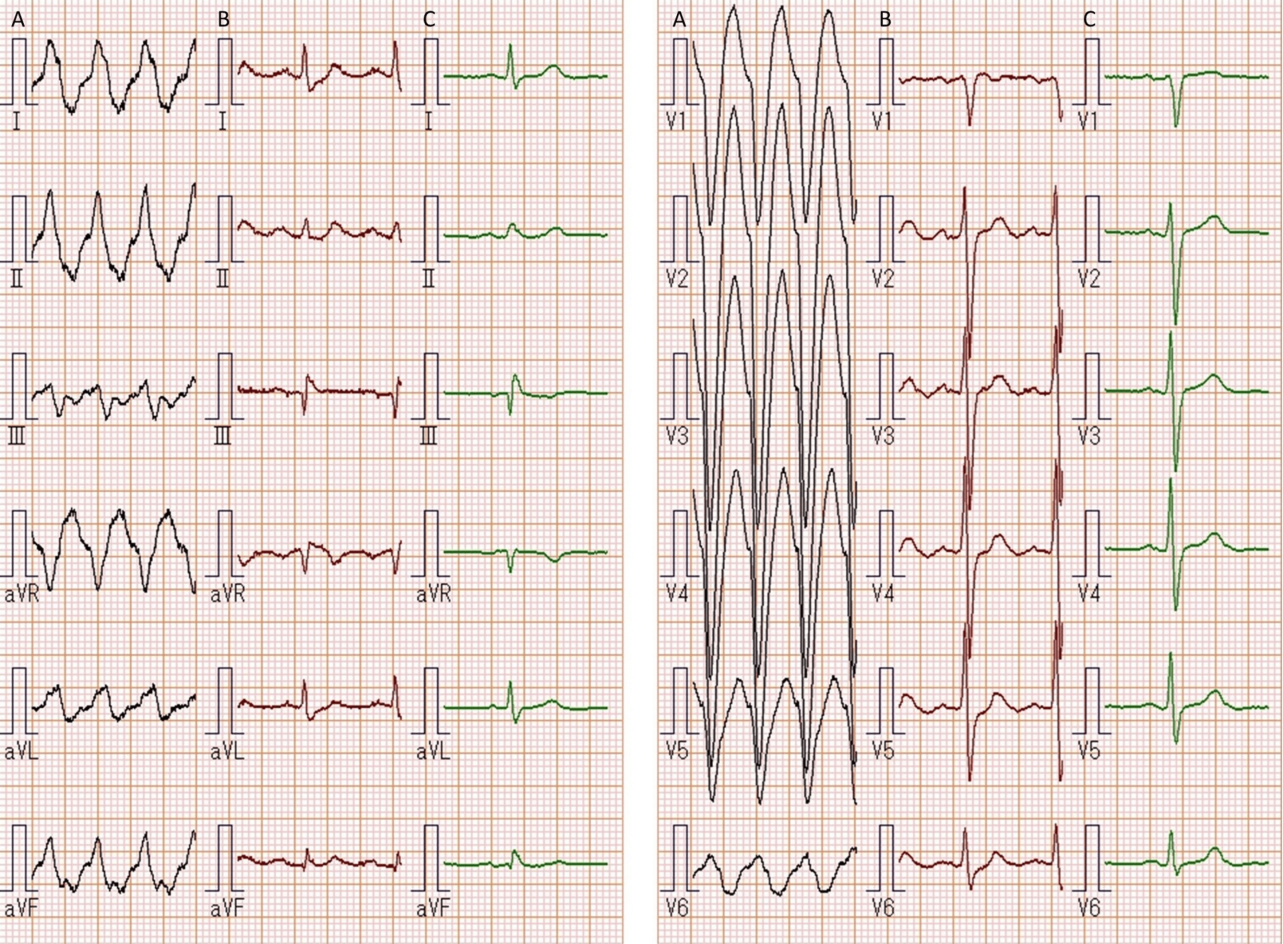
Electrocardiography On arrival, shown are a regular tachycardia of 206 beats per minute, a wide QRS of 145 ms, and an axis of 16° (A), findings consistent with a right ventricular anterolateral free wall origin. After an intravenous injection of lidocaine 100 mg, a normal sinus rhythm is obtained (B), findings consistent with the previous findings obtained approximately 10 months earlier (C).

On examination, he seemed sick. The blood pressure was 113/77 mmHg, the pulse rate was 180 beats per minute, the body temperature was 36.1°C, the respiratory rate was 30 breaths per minute, and the oxygen saturation level was 93% while he was breathing ambient air. His extremities were cold; neither jugular venous distention nor livedo reticularis was noted. No gallop was audible, and the lungs were clear on auscultation. There was no edema in his legs. Electrocardiography demonstrated a regular wide QRS tachycardia (Figure 1A). A presumed diagnosis of ventricular tachycardia was made, and a lidocaine of 100 mg was intravenously initiated. Approximately three minutes later, a normal sinus rhythm was obtained (Figure 1B), findings consistent with the previous recording obtained 10 months earlier (Figure 1C). His symptoms abated, and his vital signs returned to normal.

The liver and renal blood tests were normal, as were the complete blood cell counts and electrolyte balance. The brain natriuretic peptide level was 8.1 pg/ml (reference value, ≤18.4), and the high-sensitivity troponin T level was 0.006 ng/mL (reference value, ≤0.014). The chest radiograph was unremarkable, and bedside echocardiography was considered to show normal ventricular function and chamber sizes at this moment. Computed tomography of the heart obtained after administration of contrast material showed normal coronary arteries and cardiac structure. The patient was admitted to the intensive care unit.

**Figure 2:**
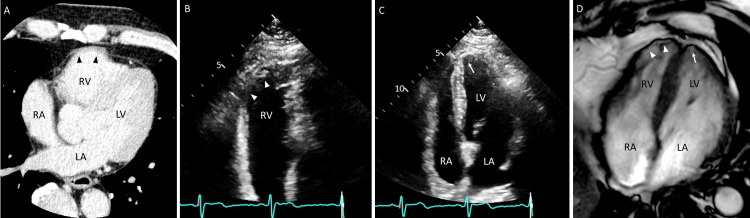
Cardiac imaging Computed tomography of the heart shows bulging of the right ventricular free (RV) wall (A, arrowheads). Repeat echocardiography shows asynergy not only in the RV free wall (B, arrowheads) but also in the left ventricular (LV) apex (C, arrow). Cardiac magnetic resonance shows partial thinning in the same regions of both RV and LV (D, arrowheads and arrow, respectively). LA indicates left atrium; RA, right atrium.

Reviewing the CT of the heart showed a bulge of the right ventricular free wall (Figure 2A), which was consistent with the origin of the sustained ventricular tachycardia (i.e., the anterolateral free wall of the right ventricle). Follow-up echocardiography revealed abnormal wall motion not only in the right ventricular free wall but also in the left ventricular apex (Figure 2B-2C). Similar findings were seen on cardiac magnetic resonance (Figure 2D), with a slightly high-intensity signal on T2-weighted images and positive findings on late gadolinium enhancement in the right ventricular free wall and left ventricular apex. On 18F-fluorodeoxyglucose positron emission tomography imaging, no abnormal tracer uptake was noted except in the heart; mild positive uptake was observed in both regions of the right ventricular free wall and the left ventricular apex.

His clinical course was uneventful, and an implantable cardioverter defibrillator was placed at another hospital. Although an endomyocardial biopsy from the right ventricle was negative, a diagnosis of isolated CS was made according to the guidelines proposed by the Japanese Circulation Society [3]. He was being considered for immunosuppressive therapy, including steroids.

## Discussion

Our patient presented with sustained ventricular tachycardia that was terminated by intravenous administration of lidocaine. After recovery, electrocardiography and echocardiography showed no abnormal findings, and cardiac computed tomography with contrast showed no coronary stenosis. However, abnormal wall motion in the free wall of the right ventricle and the apex of the left ventricle was later recognized by CT images and follow-up echocardiography. A diagnosis of isolated CS was made, and an implantable cardioverter defibrillator was placed at another hospital. The patient was being scheduled for steroid treatment.

Sarcoidosis is a disease of unknown etiology characterized by non-caseating granulomas, with an annual incidence ranging from 1-3 per 100,000 population in Asians and Hispanics to 17-35 per 100,000 population in African Americans, typically between 35 and 50 years of age at diagnosis in different populations [4]. Although sarcoidosis can affect any organ, pulmonary sarcoidosis (i.e., the lungs and intrathoracic lymph nodes) is the most common manifestation (>90% of patients) [5]. Affected sites of extrapulmonary sarcoidosis include the skin (e.g., cutaneous sarcoidosis), eyes (e.g., uveitis), joints (e.g., acute oligoarthritis), liver, gastrointestinal tract, nervous system, kidneys (e.g., granulomatous interstitial nephritis), and heart.

The prevalence of CS varies widely, from 1% to 70%, probably due to methodological differences and diagnostic difficulties [4]. A retrospective study of 110 patients diagnosed with histologically confirmed CS in Finland showed that 65% had clinically isolated CS, defined here as cardiac involvement with no history or current signs or symptoms of extracardiac sarcoidosis on initial clinical examination, routine blood tests, and plain chest X-ray [6]. The initial clinical manifestations in all 110 patients with CS and 71 patients with isolated CS were symptomatic atrioventricular conduction block (44% and 48%, respectively), ventricular tachycardia or fibrillation (33% and 38%), and heart failure (18% and 11%) [6]. The exact mechanism of sustained ventricular tachycardia remains unclear in our patient, but myocardial scar associated with inflammatory sarcoid granulomas has been reported to be the dominant substrate for this condition [7]. Given that the initial symptom in patients with CS can be fatal, screening for cardiac involvement is paramount in all patients diagnosed with sarcoidosis and in those suspected of having sarcoidosis.

The initial examination for possible cardiac involvement in sarcoidosis is electrocardiography and echocardiography. In electrocardiography, in addition to first-degree and high-degree atrioventricular block, QRS complex (e.g., fragmented QRS complex and bundle branch block), QT interval (e.g., increased QT dispersion), and T-wave characteristics are considered important in identifying patients at risk for cardiac involvement [8], none of which were observed in the current patient's electrocardiography after return to sinus rhythm. Our patient was also initially considered to have normal echocardiographic findings in the emergency department, but caution should be paid because there appear to be factors that alter echocardiographic assessment. For example, releasing catecholamines in an emergency may increase the risk of missing a wall motion abnormality in a limited segment, as in our case.

It is important to recognize that the possibility of CS cannot be ruled out by normal electrocardiography and echocardiography. In a study of 112 consecutive patients with biopsy-proven extracardiac sarcoidosis, the prevalence of CS was 18% in patients with normal findings on both electrocardiography and transthoracic echocardiography [9]. The low sensitivity of the initial screening tests, as well as the lack of specific biomarkers, may lead to the underdiagnosis of CS in clinical practice. Since endomyocardial biopsy has a relatively low yield (~25%), although it is considered the gold standard for establishing the diagnosis of CS [1], surrogate cardiac imaging is required for diagnosis, as shown in our case. As CS is reported to be a leading cause of mortality in patients with sarcoidosis (i.e., 85% die from cardiac complications) [10], not only careful assessment of fundamental examination, including electrocardiography and echocardiography, but also more use of sophisticated medical modalities, including cardiac magnetic resonance and 18F-fluorodeoxyglucose positron emission tomography, may be required in selected patients to detect cardiac involvement.

## Conclusions

We presented a case of sustained ventricular tachycardia thought to originate from the anterolateral free wall of the right ventricle, leading to the diagnosis of isolated CS, although not pathologically proven. Our case highlights the importance of careful evaluation of cardiac images and passive adaptation of more sophisticated cardiac modalities for the detection of CS.
